# Emerging Roles of Immune Cells in Cancer Development and Progression

**DOI:** 10.3390/cancers14112642

**Published:** 2022-05-26

**Authors:** David Entenberg, Panagiota S. Filippou, George S. Karagiannis

**Affiliations:** 1 Department of Pathology, Albert Einstein College of Medicine/Montefiore Medical Center, Bronx, New York, NY 10461, USA; 2 Integrated Imaging Program, Albert Einstein College of Medicine/Montefiore Medical Center, Bronx, New York, NY 10461, USA; 3 Gruss-Lipper Biophotonics Center, Albert Einstein College of Medicine/Montefiore Medical Center, Bronx, New York, NY 10461, USA; 4 Tumor Microenvironment and Metastasis Program, Albert Einstein Cancer Center, Bronx, New York, NY 10461, USA; 5 School of Health & Life Sciences, Teesside University, Middlesbrough TS1 3BX, UK; 6 National Horizons Centre, Teesside University, 38 John Dixon Ln, Darlington DL1 1HG, UK; 7 Department of Microbiology & Immunology, Albert Einstein College of Medicine, Bronx, New York, NY 10461, USA

Immune cells are a major constituent of the tumor microenvironment, and participate in interactions with tumor cells to promote the acquisition of critical hallmarks of cancer [1,2]. Myeloid cells (e.g., macrophages, neutrophils, and myeloid-derived suppressor cells) contribute significantly to the regulation of the critical steps of the metastatic cascade [3,4]. Moreover, cancer cells evade immune destruction through the development of molecular and cellular strategies, turning both innate (e.g., natural killer (NK) cells) and adaptive (CD8+ T lymphocytes) immune cells into unwitting supporters of the metastatic process [5]. In recent years, there have been many studies undertaken exploring the mechanisms underpinning the role of immune cells in cancer metastasis and progression [6]. A thorough understanding of the mechanisms of pathobiology is paramount for the development of novel and efficient immunotherapies [7], capable of specifically targeting pre-metastatic immune cell functions. This collection of articles attempts to dissect the molecular and cellular mechanisms by which immune cells regulate cancer metastasis and progression. These efforts also provide insight into novel microenvironment-based targets that may lead to the development of rationalized and personalized cancer immunotherapy.

A specific subtype of myeloid cells, the Tie2-expressing macrophage, has recently received attention due to its critical role in both tumor angiogenesis and the dissemination of tumor cells from primary tumors to secondary sites. The angiopoietin–Tie2 system is an appropriate pharmacological target within the tumor microenvironment, able to disrupt angiogenesis and metastasis. Duran et al. present an elegant review of the complex effects the Ang–Tie2 signaling axis has in the context of reciprocal interactions among endothelial cells, immune cells, and tumor cells. The authors conclude that this signaling axis is a critical milestone in designing therapies to improve the survival of patients with metastatic disease [8].

Natural killer and dendritic cells (DCs) have also attracted attention, since the former play a key role in tumor immunosurveillance by directly killing malignant cells, while the latter link innate and adaptive immune systems by cross-presenting antigens to T cells [1]. However, the presence of an immunosuppressive tumor milieu can either lead to complete inactivation or the poor function of NKs and DCs, resulting in an adverse outcome for cancer patients. In this regard, Ahluwalia et al. review the biological roles and prognostic and predictive aspects of NK and DC cells, and present the newly developed immunotherapy strategies that have the potential to enhance their antitumor function in non-small cell lung carcinoma [9]. Although recent strategies aiming to activate DC-based immunity through vaccination look promising, the immunosuppressive microenvironment is still a major barrier. As such, the authors propose that novel strategies to understand and utilize critical mediators of DC function would benefit cancer patients. Similarly, the durability of NK cell-based approaches suffers from ineffective activation, a lack of tumor-specific NK cells, and/or due to a low mutational burden in tumor cells. In this review, the authors suggest that immunotherapies based on DCs and NK cells can be combined with other therapies to maximize clinical benefit [9].

Antitumor immune surveillance is overseen by CD8+ T lymphocytes primed by specific DCs. Therefore, a high level of tumor infiltration of CD8+ cytotoxic T lymphocytes (as well as the presence of adjacent tertiary lymphoid structures) is associated with a favorable prognosis in cancer patients [10]. One of the hallmarks of cancer, “evading immune surveillance”, is accomplished by inhibiting T cell effector functions [2], for instance, by upregulating checkpoint inhibitors such as the programmed cell death-1/programmed death ligand-1 (PD-1/PD-L1) pathway. To determine the translational promise and prognostic value of the PD-1/PD-L1 pathway, Wirta et al. evaluate immune cell infiltration and PD1/PD-L1 expression in a cohort of patients with small bowel adenocarcinoma (SBA) that had previously been verified to have predisposing conditions, as well as the exome-wide characterization of somatic mutations [11]. The study determines that combining an immune cell score (ICS), formed from the number of CD8+ lymphocytes in the tumor, with PD-L1IC and PD-1 forms a new metric, here called “immunoprofile”, that is prognostic for better patient outcome [11].

Tumor-associated neutrophils (TANs) constitute another important portion of infiltrating immune cells in the tumor micro-environment [12], exerting either pro-tumor (N2 TANs) or anti-tumor (N1 TANs) properties. Recent studies on neutrophils have drawn attention to the pro-tumorigenic role of neutrophil extracellular traps (NETs) in the tumor microenvironment. Recent developments in this aspect are provided in a concise review by Chen et al., examining the pivotal roles of NETs in the metastatic cascade and their potential harnessing in clinical applications. Mechanistically, NETs are involved in supporting local invasion, vascular permeability, and immune escape, thus, promoting tumor metastasis [13]. Translationally, NETs have great potential to guide clinical diagnosis and prognosis as cancer biomarkers, or if treated as therapeutic targets to prevent metastasis [13].

TANs are recruited in the tumor microenvironment via specific neoplastic signals to exert either tumor-promoting or tumor-suppressive effects. Chemokines in the tumor microenvironment are essential for the control of tumor progression. As neutrophils are also increasingly recognized as key modulators of tumor progression, and since they highly express the chemokine receptor Cxcr2, Timaxian et al. performed an animal study showing the loss of Cxcr2 expression to have a pro-tumorigenic effect, leading to an increased primary tumor growth and lung metastases [14]. By performing a transcriptomic analysis, they show that Cxcr2−/− TANs have a more pronounced TAN2 phenotype than WT TANs with multiple pathways of neutrophil action dysregulated, suggesting that Cxcr2 could be involved in TAN plasticity [14]. Moreover, Cxcr2 ablation alters neutrophil properties, with Cxcr2−/− TANs showing a reduced anti-tumor ability [14], thus, reinforcing the importance of Cxcr2 in neutrophil function in cancer progression.

Kuziel et al. explore the role of CCL2 in macrophage-induced inflammation and breast tumor pathology. They suggest that obesity-driven chronic inflammation induced by CCL2 significantly enhances tumor growth and promotes the formation of a desmoplastic stroma through the early recruitment of macrophages and fibrocytes in the tumor microenvironment [15]. Thus, fibrocytes may be a novel target in the tumor microenvironment to reduce tumor fibrosis and enhance treatment responses for obese breast cancer patients. Furthermore, understanding how macrophages contribute to cancer-associated fibroblast (CAF) formation during early tumor progression may lead to novel chemoprevention strategies to reduce breast tumor growth and progression for at-risk obese women [15]. Myeloid-derived suppressor cells (MDSCs) represent a heterogeneous subset of myeloid cells with immunosuppressive properties, capable of sustaining the metastatic process [16]. MDSCs are able to both suppress immune cells (e.g., T and natural killer) and enhance other immunosuppressive cells (e.g., regulatory T (Treg) and B cells (Breg)), as well as produce a number of immunosuppressive molecules (e.g., IL-10 and TGF- β) [17]. In this regard, Zarobkiewicz et al. investigate the potential role of MDSCs in hematopoietic malignancies by correlating the frequency of circulating monocytic MDSCs (M-MDSC) with clinical and laboratory parameters associated with disease progression and immune status in patients with chronic lymphoid leukemia (CLL) [18]. Interestingly, the authors observe an increased percentage of M-MDSCs in CLL patients, with M-MDSC being highest in patients with adverse prognostic factors [18].

In conclusion, this collection comprises informative research and comprehensive review articles aiming to better understand the involvement of immune cells in cancer metastasis and progression, as well as their clinical and prognostic applications. Since the field is broad, the intention was not to provide an exhaustive array of pro-metastatic and immunosuppressive mechanisms elicited by immune cells onto the tumor microenvironment. Rather, we wished to highlight the complex nature of tumor-immune cell interactions by portraying the challenges and difficulties encountered when studying them mechanistically. We anticipate our collection to be successful in smoothly introducing this field to young researchers, while simultaneously inspiring more established researchers to address these challenges, with the vision of improving cancer immunotherapy and personalized medicine.

## References

[1] Hiam-Galvez K.J., Allen B.M., Spitzer M.H. (2021). Systemic immunity in cancer. Nat. Rev. Cancer.

[2] Hanahan D. (2022). Hallmarks of Cancer: New Dimensions. Cancer Discov..

[3] Swierczak A., Pollard J.W. (2020). Myeloid Cells in Metastasis. Cold Spring Harb. Perspect. Med..

[4] Sanchez L.R., Borriello L., Entenberg D., Condeelis J.S., Oktay M.H., Karagiannis G.S. (2019). The emerging roles of macrophages in cancer metastasis and response to chemotherapy. J. Leukoc. Biol..

[5] Kitamura T., Qian B.Z., Pollard J.W. (2015). Immune cell promotion of metastasis. Nat. Rev. Immunol..

[6] Garner H., de Visser K.E. (2020). Immune crosstalk in cancer progression and metastatic spread: A complex conversation. Nat. Rev. Immunol..

[7] Asiry S., Kim G., Filippou P.S., Sanchez L.R., Entenberg D., Marks D.K., Oktay M.H., Karagiannis G.S. (2021). The Cancer Cell Dissemination Machinery as an Immunosuppressive Niche: A New Obstacle Towards the Era of Cancer Immunotherapy. Front. Immunol..

[8] Duran C.L., Borriello L., Karagiannis G.S., Entenberg D., Oktay M.H., Condeelis J.S. (2021). Targeting Tie2 in the Tumor Microenvironment: From Angiogenesis to Dissemination. Cancers.

[9] Ahluwalia P., Ahluwalia M., Mondal A.K., Sahajpal N.S., Kota V., Rojiani M.V., Kolhe R. (2021). Natural Killer Cells and Dendritic Cells: Expanding Clinical Relevance in the Non-Small Cell Lung Cancer (NSCLC) Tumor Microenvironment. Cancers.

[10] Fridman W.H., Zitvogel L., Sautes-Fridman C., Kroemer G. (2017). The immune contexture in cancer prognosis and treatment. Nat. Rev. Clin. Oncol..

[11] Wirta E.V., Szeto S., Hänninen U., Ahtiainen M., Böhm J., Mecklin J.P., Aaltonen L.A., Seppälä T.T. (2020). Prognostic Value of Immune Environment Analysis in Small Bowel Adenocarcinomas with Verified Mutational Landscape and Predisposing Conditions. Cancers.

[12] Burn G.L., Foti A., Marsman G., Patel D.F., Zychlinsky A. (2021). The Neutrophil. Immunity.

[13] Chen Q., Zhang L., Li X., Zhuo W. (2021). Neutrophil Extracellular Traps in Tumor Metastasis: Pathological Functions and Clinical Applications. Cancers.

[14] Timaxian C., Vogel C.F.A., Orcel C., Vetter D., Durochat C., Chinal C., Nguyen P., Aknin M.L., Mercier-Nome F., Davy M. (2021). Pivotal Role for Cxcr2 in Regulating Tumor-Associated Neutrophil in Breast Cancer. Cancers.

[15] Kuziel G., Thompson V., D'Amato J.V., Arendt L.M. (2020). Stromal CCL2 Signaling Promotes Mammary Tumor Fibrosis through Recruitment of Myeloid-Lineage Cells. Cancers.

[16] Cole K., Pravoverov K., Talmadge J.E. (2021). Role of myeloid-derived suppressor cells in metastasis. Cancer Metastasis Rev..

[17] Groth C., Hu X., Weber R., Fleming V., Altevogt P., Utikal J., Umansky V. (2019). Immunosuppression mediated by myeloid-derived suppressor cells (MDSCs) during tumour progression. Br. J. Cancer.

[18] Zarobkiewicz M., Kowalska W., Chocholska S., Tomczak W., Szymanska A., Morawska I., Wojciechowska A., Bojarska-Junak A. (2020). High M-MDSC Percentage as a Negative Prognostic Factor in Chronic Lymphocytic Leukaemia. Cancers.

